# Artificial intelligence driven intraocular lens power calculation in extreme axial myopia

**DOI:** 10.1038/s41598-025-20899-6

**Published:** 2025-10-22

**Authors:** Yudai Suzuki, Koju Kamoi, Kengo Uramoto, Kyoko Ohno-Matsui

**Affiliations:** 1 Department of Ophthalmology & Visual Science Graduate school of Medical and Dental Sciences , Institute of Science Tokyo , Tokyo, Japan; 2 Department of Ophthalmology, Kawaguchi Municipal Medical Center, Saitama, Japan

**Keywords:** Extreme axial myopia, AI-driven formulas, Machine learning, Refractive outcomes, Hyperopic shifts, Optical physics, Eye diseases

## Abstract

Accurate intraocular lens (IOL) power calculation is critical in cataract surgery, especially in patients with extreme axial myopia where traditional formulas often yield inaccurate results. This study retrospectively evaluated the accuracy of two AI-driven IOL formulas (Hill-RBF, Kane), the Barrett Universal II formula, and the traditional SRK/T formula in patients with axial lengths ≥ 30.0 mm. Data from 80 eyes of 51 patients treated at the Institute of Science Tokyo were analyzed. Postoperative refractive errors were recalculated, and accuracy was assessed using mean error (ME), mean absolute error (MAE), and median absolute error (MedAE). Statistical analyses included the Wilcoxon signed-rank test and chi-square test. The Kane and Hill-RBF formulas demonstrated significantly lower MAE (0.51 D and 0.52 D, respectively) compared to SRK/T (*P* < 0.05). MAE of the Barrett Universal II formula was 0.66D, which was not significantly different from SRK/T. In eyes with axial lengths ≥ 32.0 mm, Kane achieved the lowest MAE and MedAE (0.44 D and 0.40 D). Both Kane and Hill-RBF showed lower refractive errors > ± 1.0 D (7.5%) compared to SRK/T (42.5%). AI-driven formulas, particularly Kane and Hill-RBF, significantly improve refractive accuracy in extreme axial myopia. Their clinical adoption may enhance postoperative visual outcomes and reduce the need for corrective interventions.

## Introduction

Cataract surgery is one of the most common surgical procedures worldwide, critically dependent on precise intraocular lens (IOL) power calculations to ensure optimal postoperative visual acuity and quality of life^1^. Despite significant advancements in surgical techniques and IOL technology, achieving accurate refractive outcomes in patients with axial lengths of 28.0 mm or more remains particularly challenging^2,3^. These patients present unique optical characteristics that complicate the calculation of IOL power, often leading to significant postoperative refractive errors when traditional formulas are used^4^.

The SRK/T formula, a long-standing standard in IOL power calculation, is known for its wide applicability across various patient demographics^5^. However, it has been observed to falter in cases with extreme axial lengths, leading to less predictable outcomes and a higher likelihood of significant postoperative refractive errors^6^. This can necessitate additional surgical interventions to replace the IOL and achieve the desired visual outcomes. One major issue is the inaccurate estimation of the effective lens position (ELP), a key variable in IOL power prediction. In long eyes, the anterior segment parameters do not scale linearly with axial length, making ELP more difficult to predict using regression-based models. These factors contribute to a higher incidence of postoperative refractive surprises in long eyes, underscoring the need for more advanced, AI-driven formulas that can account for these complex, non-linear interactions.

In recent years, the Barrett Universal II formula has emerged as a more advanced option, incorporating a multifactorial approach to better account for the complexities involved in eyes with long axial lengths^7^. This formula is not AI-based but employs a theoretical eye model and uses multiple biometric parameters including lens thickness and white-to-white corneal diameter to enhance ELP prediction and overall accuracy^8^. Despite these advancements, the challenges remain, and the quest for even more precise calculation methods continues^9^.

The integration of artificial intelligence (AI) into medical practices has opened new frontiers in ophthalmology. AI-enhanced IOL calculation formulas such as Hill-RBF and Kane have been developed^10,11^, leveraging machine learning algorithms to analyze large datasets and refine predictive models. These AI-driven formulas offer enhanced accuracy by adapting to the specific anatomical and refractive properties of each patient’s eye, particularly those with abnormal axial lengths^12^. The Hill-RBF formula is based on a pattern recognition algorithm utilizing radial basis functions, trained on a large dataset of postoperative outcomes. It does not rely on traditional theoretical optics, but instead recognizes biometric patterns to predict IOL power. The Kane formula, on the other hand, integrates theoretical optics with AI-driven regression models and incorporates both biometric inputs and demographic data such as sex.

In this study, we evaluated the accuracy of four contemporary IOL power calculation formulas: two AI-driven formulas, Hill-RBF and Kane, and two non-AI formulas, Barrett Universal II and SRK/T. While this is a narrower scope compared to studies evaluating a larger number of formulas, our analysis focuses on some of the most widely used and advanced formulas currently available. The primary goal is to identify the formula that provides the most reliable and precise predictions for refractive outcomes in patients with elongated axial lengths, thereby potentially reducing the rate of postoperative complications and enhancing overall patient satisfaction with their visual results. By directly comparing these four formulas, this study provides clinically relevant insights into their performance, particularly in challenging cases of extreme axial myopia^13–15^.

## Results

### Patient characteristics and Iol characteristics

The study evaluated 80 eyes of 51 patients (16 males, 64 females; mean age 64.8 ± 7.2 years), all of whom met the inclusion criteria. The mean axial length was 31.8 ± 1.2 mm. Other biometric data are summarized in Table 1. IOL data are summarized in Table 2. Each IOL formula’s optimized A constants and corresponding metrics are presented in Table 3.

**Table 1:** Patient characteristics.

Parameter	Value
Number of cases (Patients)	80 (51)
Gender (Male/Female)	16/64
Age (years, mean ± SD)	64.8 ± 7.2 (52–79)
Axial length (mm, mean ± SD)	31.8 ± 1.2 (30.01–34.82)
Corneal curvature radius (D)	44.0 ± 1.5 (41.34–47.10)
Anterior chamber depth (mm)	3.5 ± 0.48 (2.86–5.12)
Lens thickness (mm)	4.6 ± 0.81 (3.78–6.37)
Corneal diameter (mm)	11.9 ± 0.42 (11.5–13.4)
Corneal thickness (µm)	539 ± 33 (481–576)

D Diopters, SD Standard deviation, mm millimeters, µm micrometers. Values are presented as mean ± standard deviation (SD)

**Table 2:** Summary of intraocular lens.

IOL Model	Manufacturer		Material		Design		Number of eyes		
XY-1	HOYA		Hydrophobic acrylic		Monofocal		31		
MN60MA	ALCON		Hydrophobic acrylic		Monofocal		49		

IOL Intraocular lens. Two IOL models were used: XY-1and MN60MA, both monofocal/hydrophobic acrylic lenses.

**Table 3:** Summary of each intraocular lens formula a constants and metrics.

Formula	A constants	Metrics	Optimization details
SRK/T	123.74	AL, K, ACD	Optimized using the dataset to achieve arithmetic mean ME of zero.
Barrett Universal II	121.85	AL, K, ACD, LT, WTW	Considers additional biometric parameters like lens thickness and white-to-white.
Hill-RBF	118.89	AL, K, ACD, LT, WTW, CCT	Utilizes artificial intelligence for constant refinement and prediction accuracy.
Kane	117.00	AL, K, ACD, LT, CCT, gender	Incorporates demographic factors like gender for enhanced precision.

AL = axial length, K = keratometry, ACD = anterior chamber depth, LT = lens thickness, WTW = white-to-white, CCT = central corneal thickness. Constants were optimized iteratively to minimize arithmetic mean ME and ensure comparability among formulas

### Refractive error analysis

Analysis of absolute refractive error values revealed significant differences among the IOL calculation formulas used, as shown in Table 4. The SRK/T formula showed a SD, MAE and MedAE of 1.15, 0.96 diopters (D) and 0.87 D, which was the highest among the formulas tested. In comparison, the Barrett Universal II formula had a SD, MAE and MedAE of 0.84, 0.66 D and 0.57 D, the Hill-RBF formula showed 0.63, 0.52 D and 0.43 D and the Kane formula exhibited at 0.63, 0.51 D and 0.43D. Specifically, in Group B (axial length ≥ 32.0 mm), the Hill-RBF and Kane formulas showed the lowest MAE (0.49 D and 0.44 D) and MedAE (0.43 D and 0.40 D) compared to the other formulas. The Hill-RBF formulas and Kane showed a significantly lower error compared to SRK/T in Group B (*P* < 0.05). Detailed statistical analysis is shown in Table 5.

**Table 4:** Refractive outcomes by IOL calculation formula.

Axial length group	Group A (30 ≤ AL < 32)	Group B (32 ≤ AL)	Combined
	ME/SD/MAE/MedAE	ME/SD/MAE/MedAE	ME/SD/MAE/MedAE
SRK/T	0.42/1.16/1.04/0.96	−0.46/0.99/0.87/0.77	0.00/1.15/0.96/0.87
Barrett Universal II	−0.28/0.86/0.70/0.60*	0.34/0.67/0.61/0.49	0.00/0.84/0.66/0.57*
Hill-RBF	0.07/0.68/0.54*/0.43*	−0.09/0.56/0.49*/0.43	0.00/0.63/0.52*/0.43*
Kane	0.15/0.69/0.56*/0.46*	−0.19/0.50/0.44*/0.40*	0.00/0.63/0.51*/0.43*

ME = Mean Prediction Error (D) /SD = Standard Deviation (D)/ MAE = Mean Absolute Error (D)/ MedAE = Median Absolute Error (D) D = Diopters, AL = Axial Length. Values are presented as ME/SD/MAE/MedAE (Diopters). Statistical significance was determined using the Wilcoxon rank-sum test for non-normally distributed data.

**Table 5:** Statistical analysis between groups by wilcoxon rank-sum test (MAE and MedAE).

Comparison	Group A (30 ≤ AL < 32)	Group B (32 ≤ AL)	Combined
SRK/T vs. Barrett II	MAE: 0.017515/MedAE: 0.005*	MAE: 0.1037/MedAE: 0.112	MAE: 0.0047*/MedAE: 0.0013*
SRK/T vs. Hill-RBF	MAE: 0.0005*/MedAE: 0.00004*	MAE: 0.0041*/MedAE: 0.011	MAE: 0.000005*/MedAE: 0.000002*
SRK/T vs. Kane	MAE: 0.0003*/MedAE: 0.00014*	MAE: 0.0012*/MedAE: 0.005*	MAE: 0.0000006*/MedAE: 0.000002*
Barrett II vs. Hill-RBF	MAE: 0.0708/MedAE: 0.212	MAE: 0.1866/MedAE: 0.364	MAE: 0.0248/MedAE: 0.1217
Barrett II vs. Kane	MAE: 0.1722/MedAE: 0.265	MAE: 0.0897/MedAE: 0.171	MAE: 0.0341/MedAE: 0.0836
Hill-RBF vs. Kane	MAE: 0.4800/MedAE: 0.887	MAE: 0.4642/MedAE: 0.459	MAE: 0.994/MedAE: 0.7342

MAE Mean absolute error, MedAE Median absolute error, AL Axial length Wilcoxon rank-sum test was used due to non-normal data distribution (assessed via the Kolmogorov–Smirnov test). Asterisks (*) indicate statistical significance at P < 0.05.MedAE comparisons highlight differences in the central tendency of absolute errors, while MAE reflects the mean directional error

A stratified analysis of prediction error by IOL model was performed. The results are shown in Table 6. For the MN60MA group, MAE and MedAE for each formula were as follows: SRK/T (0.86 D and 0.77 D), Barrett Universal II (0.62 D and 0.54 D), Hill-RBF (0.54 D and 0.45 D), and Kane (0.49 D and 0.41 D). In comparison, for the XY1 group, the MAE/MedAE values were: SRK/T (1.20 D and 1.19 D), Barrett Universal II (0.72 D and 0.60 D), Hill-RBF (0.48 D and 0.40 D), and Kane (0.54 D and 0.48 D). These findings suggest that both IOL models showed improved accuracy with the newer generation formulas, and that the differences between IOL groups may reflect variations in axial length rather than IOL geometry alone.

**Table 6:** Refractive outcomes by IOL type.

IOL Group	MN60MA	XY1
	MAE/MedAE	MAE/MedAE
SRK/T	0.86/77	1.20/1.19
Barrett Universal II	0.62/0.54	0.72/0.60
Hill-RBF	0.54/0.45	0.48/0.40
Kane	0.49/0.41	0.54/0.48

MAE Mean absolute error, MedAE Median absolute error

### Hyperopic shift incidence

Further analysis focused on hyperopic shifts exceeding + 1.0 D, which are clinically significant as they can affect visual outcomes post-surgery. The maximum and minimum refractive errors observed for each formula are shown in Table 7.

**Table 7:** Maximum and minimum refractive errors (D) by IOL calculation formula.

Formula	Maximum refractive error (D)	Minimum refractive error (D)
SRK/T	2.13	2.59
Barrett Universal II	2.09	2.88
Hill-RBF	2.05	1.89
Kane	1.97	1.83

D Diopters. Maximum and minimum values represent the extreme observed refractive errors for each formula.

The SRK/T formula resulted in a 4 2.5% incidence of hyperopic shifts, which was substantially higher than the other formulas: Barrett Universal II (18.8%), Hill-RBF (7.5%), and Kane (7.5%) (Table 8). This marked reduction in hyperopic errors highlights the superior performance of newer formulas in managing patients with long axial lengths. To account for inter-formula correlation within the same eyes, we additionally performed Cochran’s Q test to evaluate differences in the incidence of refractive errors ≥ ± 1.0D among the four formulas^16^. The test showed a statistically significant difference (Q = 42.56, *p* < 0.001), indicating that the proportion of large refractive errors varies across formulas.

**Table 8:** Proportions of refractive error categories for each formula.

Refractive Error Category	SRK/T (%)	Barrett II (%)	Hill-RBF (%)	Kane (%)
Less than ± 0.5D	26.3	45.0	55.0	65.0
Between ± 0.5D and ± 1.0D	31.3	36.3	37.5	27.5
More than ± 1.0D	42.5	18.8	7.5*	7.5*

D Diopters. Refractive error categories: Less than ± 0.5D, ± 0.5D to ± 1.0D, and more than ± 1.0D. Statistical significance was determined relative to SRK/T (*P < 0.05)

### Correlation with axial length

**Fig. 1:**
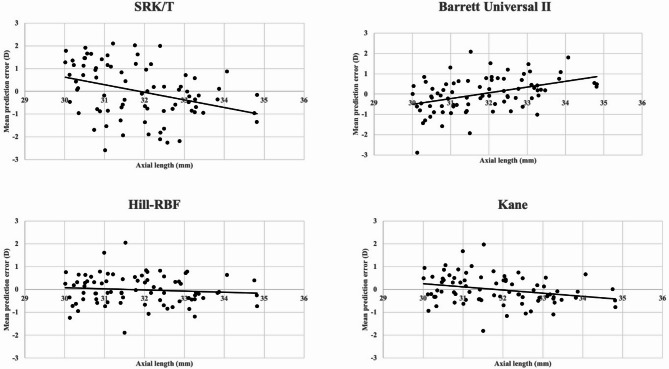
Relationship between refractive error and axial length for different iol calculation formulas. This figure illustrates the relationship between refractive error (in diopters) and axial length (in millimeters) for four different intraocular lens (IOL) calculation formulas: SRK/T, Barrett Universal II, Hill-RBF, and Kane. Each subplot represents one of the formulas and shows the spread of refractive errors across various axial lengths. The SRK/T formula demonstrates a wide spread of refractive errors, indicating higher variability and less predictability. The Barrett Universal II formula displays a tighter clustering of refractive errors, suggesting improved accuracy compared to SRK/T. The Hill-RBF and Kane formula exhibits minimal refractive errors, especially for longer axial lengths, indicating superior performance. Also, Hill-RBF and Kane formula shows the least spread of refractive errors, highlighting its high accuracy and precision in predicting postoperative refractive outcomes. Table 1 Patient characteristics.

To evaluate the relationship between axial length and refractive prediction error, Spearman’s rank correlation test was performed. The SRK/T formula showed a moderate negative correlation (ρ = − 0.381, *p* = 0.0005), indicating that prediction errors became more myopic with increasing axial length. The Kane formula also exhibited a weak negative correlation (ρ = − 0.290, *p* = 0.009), suggesting a similar trend. Barrett Universal II showed a moderate positive correlation (ρ = 0.406, *p* = 0.0002), suggesting a tendency toward hyperopic prediction errors in longer eyes. In contrast, Hill-RBF demonstrated no significant correlation (ρ = − 0.088, *p* = 0.439), implying stable refractive outcomes regardless of axial length. The relationships between refractive error and axial length for each formula are illustrated in Fig. 1.

## Discussion

Our research provides compelling evidence that the Barrett Universal II, Hill-RBF, and Kane formulas significantly outperform the traditional SRK/T formula in minimizing refractive errors in patients with elongated eyes. The superior performance of these formulas, particularly the Hill-RBF and Kane formulas, highlights the evolution of IOL power calculations from reliance on basic biometric formulas to more sophisticated data-driven approaches^17^.

The Barrett Universal II formula incorporates a multifactorial approach to account for the complex interplay of axial length, corneal power, and anterior chamber depth. This holistic view aligns with findings from previous studies, which demonstrated improved outcomes in post-operative refractive accuracy when multiple eye parameters are considered^18^. Despite these advances, our study also revealed a consistent occurrence of hyperopic shifts with the Barrett Universal II formula, corroborating observations of its limitations in eyes with extreme axial lengths^9^.

The Hill-RBF and Kane formulas leverage advanced artificial intelligence and machine learning techniques that adapt to the specific anatomical and refractive properties of each patient’s eye. The application of AI in IOL calculations allows real-time updates and learning from new data, which can improve predictive accuracy over time^19^. In particular, the Kane formula incorporates sex-specific considerations, an innovative feature that addresses demographic differences in the eye anatomy. This is supported by findings that personalized adjustments in IOL formulas could lead to better refractive outcomes, particularly in populations with a higher prevalence of myopia, such as females^20^. Of the 80 eyes analyzed, 64 were from female patients and 16 from male patients. This gender imbalance was notable and considered in the interpretation of results, particularly given that the Kane formula incorporates gender as a variable. While the Kane formula algorithmically adjusts for sex, the predominance of female patients in our cohort may have subtly favored its performance. Nevertheless, prior validations of the Kane formula have shown strong accuracy in both male and female subsets, suggesting that its superior performance in our study cannot be solely attributed to the sample’s sex distribution.

The results from Table 4 demonstrate significant differences in the mean refractive errors between groups with different axial lengths. Specifically, in Group B (32 ≤ AL), the Hill-RBF and Kane formulas showed the lower MAE (0.49 D and 0.44 D) and MedAE (0.43 D and 0.40 D) compared to the other formulas. This indicates that the Hill-RBF and Kane formulas provide more accurate predictions for patients with longer axial lengths, reducing the likelihood of significant refractive errors. The Barrett Universal II also performed better than the SRK/T formula, but not as well as the Hill-RBF formula and the Kane formula. This finding underscores the importance of selecting the appropriate formula based on axial length to achieve optimal refractive outcomes.

Table 7 shows the maximum and minimum refractive errors observed for each formula. The Kane formula demonstrated the highest accuracy with the lowest maximum refractive error (1.97D) and the highest minimum refractive error (1.83D). This further supports the Kane formula’s superior performance in managing refractive outcomes in patients with elongated axial lengths. The reduced range of refractive errors observed with the Kane formula highlights its precision and reliability.

Table 8 highlights the proportions of refractive error categories for each formula. The Kane and Hill-RBF formulas showed significantly lower proportions of cases with refractive errors greater than ± 1.0D (7.5% and 7.5%, respectively) compared to the SRK/T formula (42.5%). This reduction in significant refractive errors is critical for improving patient satisfaction and visual outcomes post-surgery. The higher proportion of cases with refractive errors less than ± 0.5D for the Kane formula (65.0%) further supports its superior accuracy in predicting IOL power. It is also important to note that while Kane and Hill-RBF demonstrated slightly better accuracy metrics in terms of mean absolute error and percentage within ± 0.5 D, these differences were not statistically significant when compared to Barrett Universal II. Thus, although the AI-enhanced formulas appear to offer advantages in certain aspects, Barrett Universal II performed comparably well and remains a reliable option in long axial length eyes.

The relationship between refractive error and axial length is illustrated in Fig. 1. The SRK/T formula showed a wide spread of refractive errors across various axial lengths, indicating higher variability and less predictability. In contrast, the Barrett Universal II formula displayed a tighter clustering of refractive errors, suggesting improved accuracy. The Hill-RBF and Kane formulas demonstrated minimal refractive errors, especially for longer axial lengths, indicating superior performance. The Hill-RBF and Kane formulas exhibited the least refractive error spread, highlighting its high accuracy and precision. These visual representations further corroborate our statistical findings and underscore the advantages of using advanced IOL calculation formulas for better refractive outcomes.

While our study provides robust evidence on the accuracy and efficacy of these formulas, it is important to acknowledge a limitation in the scope. Only four formulas were compared in this analysis, which is fewer than in some recent studies that evaluated 20 or more formulas^21^. However, these four formulas—Hill-RBF, Kane, Barrett Universal II, and SRK/T—were chosen because of their widespread clinical use and established accuracy, particularly in challenging cases of extreme axial myopia.

By evaluating these widely used formulas and comparing them against the traditional SRK/T standard, our study delivers clinically relevant insights into IOL power calculation performance in eyes with long axial lengths. Nevertheless, future studies involving a broader range of formulas and larger datasets will be essential to further validate and expand upon our findings.

Additionally, our study’s focus on a real-world clinical setting at the Institute of Science Tokyo ensures that the results are highly relevant to everyday clinical practice. Furthermore, our study highlights the importance of considering individual patient factors when selecting IOL power calculation formulas. The variability in surgical techniques and the potential biases they introduce are acknowledged, adding depth to our analysis and emphasizing the need for personalized approaches in clinical practice.

To address potential bias due to intraocular lens (IOL) model differences, we conducted a stratified analysis of refractive prediction errors by IOL type. The mean axial length for eyes implanted with MN60MA (Alcon) was 32.41 mm, whereas that for XY1 (Hoya) was 30.93 mm. Given that MN60MA is preferentially used in longer axial eyes due to its lower available power range, the observed differences in prediction accuracy may reflect axial length variations rather than IOL design alone. Although lens constant optimization was performed based on institutional data, IOL constants were not applied, which may limit generalizability^22^.

Incorporating the findings from recent studies further supports our conclusions. For instance, Stopyra analyzed twelve IOL power calculation formulas in eyes with axial myopia and found that the Barrett Universal II formula had the lowest average absolute error (AE), with the Kane formula also showing promising results for myopic eyes^12^. Additionally, a systematic review by Stopyra, Langenbucher, and Grzybowski highlighted the high precision of the Barrett Universal II, Kane, and PEARL-DGS formulas, particularly noting the superior performance of the Kane formula in long eyes^13^. Connell and Kane further confirmed the Kane formula’s lowest mean absolute prediction error across various axial lengths, supporting its superior accuracy^14^.

Other studies have consistently demonstrated the benefits of advanced formulas like the Kane and Barrett Universal II. Pereira et al. found the Kane formula to be the most accurate overall,^15^ while Savini et al. and Hipólito-Fernandes et al. both highlighted the high precision of the Kane formula among others^23,24^. Additionally, Cheng et al. reported that AI-based formulas, including Kane and Hill-RBF, significantly reduced median absolute errors in refractive predictions compared to traditional formulas^25^.

Chen et al. and Li et al. further demonstrated the effectiveness of new-generation formulas in long axial lengths, with the Kane formula consistently performing well^2,6^. Wang et al. conducted a meta-analysis confirming the superior accuracy of the Barrett Universal II in long eye^3^, while Kane et al. found the Barrett Universal II and Hill-RBF formulas to have the lowest mean absolute prediction errors^26^.

The superior performance of the Kane and Hill-RBF formulas in eyes with long axial lengths may be attributed to their ability to address two fundamental challenges in IOL power prediction: the accurate estimation of effective lens position (ELP) and the modeling of non-linear relationships between ocular biometry and refractive outcomes. The Hill-RBF formula, based on a pattern recognition algorithm trained on a large dataset of clinical cases, uses radial basis functions to interpolate across complex biometric profiles. This allows it to recognize atypical anatomical patterns that are common in long eyes, such as deeper anterior chambers or flatter corneas. In contrast, the Kane formula integrates both theoretical optics and artificial intelligence, and uniquely incorporates demographic variables like sex, which may influence eye structure. Additionally, it includes parameters such as central corneal thickness and lens thickness, which improve ELP estimation by providing more individualized eye modeling. These features likely contribute to the improved predictive accuracy observed in our cohort and support the clinical utility of AI-driven formulas for eyes with extreme axial myopia.

Li et al. reported that “Hill-RBF and Kane formulas seem to be a better choice for eyes with extremely long axial length^27^, and similar conclusions were drawn by Migi et al., who found Kane to be the better consistent performer across the axial myopia^28^.

Our findings align with this growing body of evidence and underscore the clinical utility of AI-enhanced formulas in long eyes. By incorporating sophisticated modeling and machine learning techniques, these formulas provide refractive surgeons with more reliable tools for preoperative planning in myopic eyes.

### Limitation

Our study’s limitations are noted in the context of the small sample size and short postoperative evaluation period. These factors may affect the long-term assessment of refractive outcomes, as extended follow-up periods are necessary to fully appreciate the stability of refractive errors post-surgery^29^. A power analysis based on repeated-measures ANOVA (with α = 0.05, power = 0.80, and a medium effect size of f = 0.25) indicated that approximately 179 eyes would be required to detect statistically significant differences among the four IOL formulas. Our cohort of 80 eyes falls short of this threshold, which may reduce the statistical power and increase the risk of Type II error. Furthermore, in subgroup analyses of eyes with axial length ≥ 32 mm, the small sample size further limits generalizability. Future multicenter studies with larger sample sizes are warranted to confirm and expand upon our findings. Additionally, variability in surgical techniques, as observed in our study, can introduce biases, which has been shown to significantly influence IOL power accuracy^8^. Also, although all cases involved hydrophobic acrylic monofocal IOLs, two different models were used. Minor differences in haptic design or axial stability may have influenced the effective lens position (ELP) and contributed to variability in refractive outcomes. Future studies should consider standardizing IOL type or statistically controlling for IOL design to reduce this potential confounder.

Another notable limitation is that our study compared only four formulas—SRK/T, Barrett Universal II, Hill-RBF, and Kane. While these formulas are among the most widely used and advanced options, recent studies have evaluated a broader range of formulas, including up to 20or more. This narrower scope may limit the comprehensiveness of our findings. However, the formulas selected in this study were chosen based on their established clinical relevance and widespread application in managing long axial lengths. Additionally, A key limitation of this study is that axial length adjustment was not applied to the SRK/T formula. This is known to improve its predictive accuracy in long eyes^23^,and its absence may have negatively affected SRK/T performance in our cohort. Future studies should incorporate such adjustments to ensure a more balanced comparison.

One limitation of this study is that both eyes were included for some patients, which may violate the assumption of independence in statistical analysis. We did not apply statistical adjustments such as generalized estimating equations (GEE) or bootstrap methods; therefore, the inclusion of bilateral eyes could have led to an underestimation of variance and a potential inflation of Type I error^16^. This limitation has been reported in previous literature, and should be considered when interpreting the results.

Furthermore, while our findings advocate the adoption of advanced formulas in clinical practice, it is important to consider individual patient factors when selecting IOL power calculation formulas. This includes considerations such as corneal astigmatism and previous ocular surgeries, which can affect the accuracy of IOL power predictions. The need for individualized approaches in IOL calculation was underscored by findings suggesting that tailored algorithms could potentially minimize the need for postoperative refractive corrections^30^.

A limitation of this study is the absence of multivariate analysis, which would have helped clarify the independent effects of biometric variables such as axial length, anterior chamber depth, lens thickness, and white-to-white. Due to the relatively small sample size and multicollinearity concerns, we could not include this analysis, but future studies should investigate this aspect.

## Conclusions

Our study confirms the enhanced performance of newer IOL calculation formulas, particularly the AI-driven Kane and Hill-RBF formulas, in achieving optimal refractive outcomes in patients with extreme elongated eyes. Specifically, the Hill-RBF and Kane formula demonstrated the lowest mean refractive error for axial lengths of 32.0 mm or greater. As we continue to integrate and refine these advanced algorithms, ongoing research and validation are critical to further enhance accuracy and patient satisfaction in cataract surgery. Future studies should focus on larger sample sizes, longer follow-up durations, and the inclusion of diverse surgical settings to validate and possibly expand the application of these findings across different patient populations.

## Methods

This study was a retrospective review conducted at the Institute of Science Tokyo, assessing cataract surgery outcomes from September 2020 to August 2023. The study cohort included 51 patients, totaling 80 eyes, each with an axial length exceeding 30.0 mm, as measured using the advanced Carl Zeiss IOL Master 700^23^. The inclusion criteria were strictly limited to patients who had accessible postoperative subjective refraction data one month after surgery. Patients with ocular comorbidities that could affect axial length measurement or visual outcomes were excluded. Specifically, we excluded eyes with any previous refractive surgery, corneal abnormalities, posterior staphyloma, retinal detachment, glaucoma, or any conditions that might interfere with accurate IOL power calculation^24^.

Postoperatively, the predicted refractive errors for each eye were recalculated using the SRK/T, Barrett Universal II, Hill-RBF, and Kane formulas. These predictions were then compared with actual postoperative refractive outcomes to assess the accuracy of each formula. The primary outcome measures included the mean prediction error (ME), standard deviation (SD), mean absolute error (MAE), and median absolute error (MedAE). MAE was chosen as the primary metric for formula accuracy because it reflects the average magnitude of prediction errors and is widely used in similar studies. MedAE was included as an additional metric to minimize the influence of outliers. While RMSAE was considered, it was not used as it is less commonly reported in this context. The study utilized two intraocular lens (IOL) models: XY-1 (HOYA) and MN60MA (ALCON), both monofocal and hydrophobic acrylic lenses. These models were chosen due to their widespread clinical use and compatibility with the formulas analyzed. Each formula constant was optimized for the entire dataset of patients to achieve an arithmetic mean ME of zero, ensuring comparability across formulas. The optimization process involved iterative recalibration of constants based on refractive outcomes until a mean ME of zero was achieved.

Statistical analysis was performed using SPSS software. Descriptive statistics, including the mean and standard deviation, were computed for the refractive error of each formula. Data normality was assessed by the Kolmogorov–Smirnov test. To test for statistically significant differences between the formulas, the Wilcoxon signed-rank test was employed, with Bonferroni correction applied to adjust for multiple comparisons^25^. Additionally, the chi-square test was used to analyze the incidence of significant hyperopic shifts, defined as a postoperative refractive error greater than + 1D^6^. Additionally Cochran Q test was performed as a sensitivity analysis. Statistical significance was set at *P* < 0.05. These statistical tests allowed for a robust comparison of the formula efficacy in predicting IOL power in eyes with long axial lengths.

The study protocol conformed to the tenets of the Declaration of Helsinki and was reviewed and approved by the Institutional Ethics Committee of Institute of Science Tokyo (IRB No. M2023-136). A waiver of informed consent was approved by the Ethics Committee of Institute of Science Tokyo due to the retrospective nature of the study.

## Data Availability

The datasets used and/or analysed during the current study available from the corresponding author on reasonable request.
